# 2,4 Dinitrophenol as Medicine

**DOI:** 10.3390/cells8030280

**Published:** 2019-03-23

**Authors:** John G. Geisler

**Affiliations:** Mitochon Pharmaceuticals, Inc., 970 Cross Lane, Blue Bell, PA 19422, USA; jgeisler@mitochonpharma.com; Tel.: +1-215-589-5610

**Keywords:** mitochondrial uncoupler, 2,4-dinitrophenol (DNP), Brain-derived neurotrophic factor (BDNF), neurodegeneration, anti-aging, Huntington’s Disease, Multiple Sclerosis, Duchenne Muscular Dystrophy, Traumatic Brain Injury, metabesity

## Abstract

In the sanctity of pure drug discovery, objective reasoning can become clouded when pursuing ideas that appear unorthodox, but are spot on physiologically. To put this into historical perspective, it was an unorthodox idea in the 1950’s to suggest that warfarin, a rat poison, could be repositioned into a breakthrough drug in humans to protect against strokes as a blood thinner. Yet it was approved in 1954 as Coumadin^®^ and has been prescribed to billions of patients as a standard of care. Similarly, no one can forget the horrific effects of thalidomide, prescribed or available without a prescription, as both a sleeping pill and “morning sickness” anti-nausea medication targeting pregnant women in the 1950’s. The “thalidomide babies” became the case-in-point for the need of strict guidelines by the U.S. Food & Drug Administration (FDA) or full multi-species teratogenicity testing before drug approval. More recently it was found that thalidomide is useful in graft versus host disease, leprosy and resistant tuberculosis treatment, and as an anti-angiogenesis agent as a breakthrough drug for multiple myeloma (except for pregnant female patients). Decades of diabetes drug discovery research has historically focused on every possible angle, except, the energy-out side of the equation, namely, raising mitochondrial energy expenditure with chemical uncouplers. The idea of “social responsibility” allowed energy-in agents to be explored and the portfolio is robust with medicines of insulin sensitizers, insulin analogues, secretagogues, SGLT2 inhibitors, etc., but not energy-out medicines. The primary reason? It appeared unorthodox, to return to exploring a drug platform used in the 1930s in over 100,000 obese patients used for weight loss. This is over 80-years ago and prior to Dr Peter Mitchell explaining the mechanism of how mitochondrial uncouplers, like 2,4-dinitrophenol (DNP) even worked by three decades later in 1961. Although there is a clear application for metabolic disease, it was not until recently that this platform was explored for its merit at very low, weight-neutral doses, for treating insidious human illnesses and completely unrelated to weight reduction. It is known that mitochondrial uncouplers specifically target the entire organelle’s physiology non-genomically. It has been known for years that many neuromuscular and neurodegenerative diseases are associated with overt production of reactive oxygen species (ROSs), a rise in isoprostanes (biomarker of mitochondrial ROSs in urine or blood) and poor calcium (Ca^2+^) handing. It has also been known that mitochondrial uncouplers lower ROS production and Ca^2+^ overload. There is evidence that elevation of isoprostanes *precedes* disease onset, in Alzheimer’s Disease (AD). It is also curious, why so many neurodegenerative diseases of known and unknown etiology start at mid-life or later, such as Multiple Sclerosis (MS), Huntington Disease (HD), AD, Parkinson Disease, and Amyotrophic Lateral Sclerosis (ALS). Is there a relationship to a buildup of mutations that are sequestered over time due to ROSs exceeding the rate of repair? If ROS production were managed, could disease onset due to aging be delayed or prevented? Is it possible that most, if not all neurodegenerative diseases are manifested through mitochondrial dysfunction? Although DNP, a historic mitochondrial uncoupler, was used in the 1930s at high doses for obesity in well over 100,000 humans, and so far, it has never been an FDA-approved drug. This review will focus on the application of using DNP, but now, repositioned as a potential disease-modifying drug for a legion of insidious diseases at much lower and paradoxically, weight *neutral* doses. DNP will be addressed as a treatment for “metabesity”, an emerging term related to the global comorbidities associated with the over-nutritional phenotype; obesity, diabetes, nonalcoholic steatohepatitis (NASH), metabolic syndrome, cardiovascular disease, but including neurodegenerative disorders and accelerated aging. Some unexpected drug findings will be discussed, such as DNP’s induction of neurotrophic growth factors involved in neuronal heath, learning and cognition. For the first time in 80’s years, the FDA has granted (to Mitochon Pharmaceutical, Inc., Blue Bell, PA, USA) an open Investigational New Drug (IND) approval to begin rigorous clinical testing of DNP for safety and tolerability, including for the first ever, pharmacokinetic profiling in humans. Successful completion of Phase I clinical trial will open the door to explore the merits of DNP as a possible treatment of people with many truly unmet medical needs, including those suffering from HD, MS, PD, AD, ALS, Duchenne Muscular Dystrophy (DMD), and Traumatic Brain Injury (TBI).

## 1. The Big Picture

Mitochondria are in every human cell, except mature red blood cells (RBCs), living symbiotically within the cell, having their own DNA, dividing independently by fission, growing by fusion, or culled by mitophagy, using both proteins encoded by nuclear genes or their own DNA to support their structure and function. They are essential for the life of a cell, by generating ATP from the foods we eat. Mitochondria have many more functions that are less appreciated, such as the storage depot of calcium to keep the cytosolic calcium levels low [1]. They are the only organelle with a basic pH environment due to the unique proton pumps that generate ~1 pH difference inside-out and thereby generating the proton electrochemical gradient ($\Delta {\tilde{u}}_{H^{+}}$) powering ATP production [2]. All the other cellular organelles (nucleus, lysosomes, peroxisomes) and cytosolic space have pH acidic environments. Therefore, if a molecule is cation like ethidium bromide (EtBr) or calcium (Ca^2+^) or weak acids that are transporters of a hydrogen (H^+^) cation like mitochondrial chemical uncouplers, then they preferentially target the mitochondria (101 chemistry says, “acids go to base”) [3]. In fact, although EtBr is commonly used in molecular biology as a fluorescent intercalating stain of DNA, in mitochondrial bioenergetics, low doses promote preferential damage of mitochondrial DNA and subsequently mitophagy over nuclear DNA damage. EtBr is therefore used as a tool for this purpose in bioenergetic studies to see the effects of a lower mitochondrial population on the cell’s homeostasis [3,4,5]. Mitochondrial bioenergetics are well conserved among mammals, so a drug “effect” on the mouse’s mitochondria should have a similar effect on human mitochondria [6]. In a normal functioning cell, the mitochondria population is largely homogenous. In good health, ROS production is low, ATP production high, free Ca^2+^ is low in the cytosol and Ca^2+^ storage in the mitochondria is high, but under control. There is a threshold for Ca^2+^ storage capacity. A cell in a state of mitochondrial dysfunction has a mosaic of mitochondrial capacity. Under mitochondrial dysfunction, ATP production may be variable, and ROS production higher. Diseases associated with unfolded protein response (UPR) can cause endoplasmic reticulum (ER) stress and cause the ER to flood the cytosol with Ca^2+^ [7,8]. This raised free Ca^2+^ in the cytosol, is transported by the “Ca^2+^ uniporter” into the mitochondrial matrix. Calcium levels can exceed storage capacity, inducing the mitochondrial permeability transition pore (mPTP) formation, allowing toxic mitochondrial matrix molecules to leak into the cytosol to activate programmed cell death apoptotic pathways (as seen in HD, TBI, Wolfram Syndrome, DMD, epilepsy, PD, etc.) [9,10,11,12,13,14,15]. This sets up a cascade of mitochondrial destruction since neighboring mitochondria are obligated to take up Ca^2+^, but are already at threshold capacity, so they then open the mPTP and eventually the cell (neuron, myotube, etc.) undergoes apoptosis [16]. Some diseases in particular, such as epilepsy, DMD, TBI, and those associated with misfolded proteins (PD, AD, HD, etc.) are associated with ER stress and Ca^2+^ overload.

When ROS form as a byproduct of respiration under normal conditions, the natural anti-oxidant defense mechanism keeps them safely at low levels. When there is a large production of ROS, they attack everything in the cell, including proteins, DNA and lipids. When ROS attack lipids, a double bond is formed and can easily be detected in a subpopulation of prostaglandin-like molecules called isoprostanes [17]. Isoprostanes can be measured in tissues, but are particularly relevant to clinical studies because they can be non-invasively monitored in blood and urine and are a known biomarker of ROSs and mitochondrial dysfunction in a multitude of human diseases, such as HD, Rett Syndrome, epilepsy, AD, PD, DMD/BMD, polycystic kidney diseases, etc. [18,19,20,21,22,23,24]. Drugs that lower ROSs should lower isoprostanes in urine/blood as a biomarker of resolving cellular stress. Since the mitochondrial physiology is central to many cellular functions such as governing the production of energy for the cell, storage location of Ca^2+^ for proper cell signaling, governing their own number per cell by fission/fusion, etc., but also generate the majority of the ROS, it would suggest that a dysfunctional mitochondrial population could be highly detrimental to the cell. Therefore, a healthy mitochondrial “population” is of utmost importance. The physiology of the cell and poor functioning mitochondria could be behind many diseases of known and unknown etiology since they impact so many cellular pathways.

## 2. The Pleiotropic Effects of DNP and Other Uncouplers

**Figure 1:**
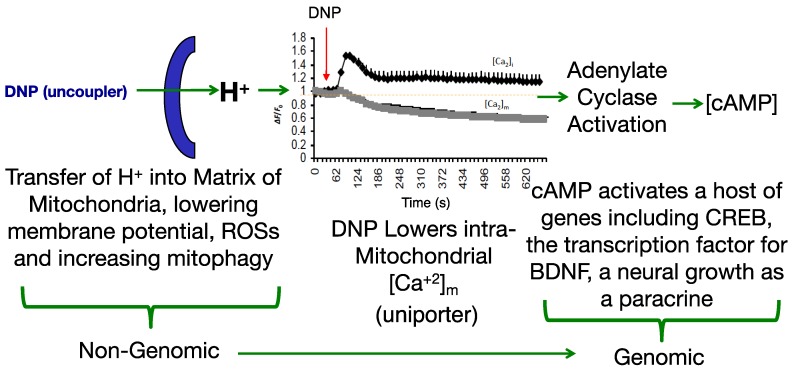
Non-genomic to Genomic Effects of 2,4-Dinitrophenol (DNP). The early immediate effects of DNP lower the mitochondrial membrane potential, which abolishes overt reactive oxygen species (ROS) production and subsequently closes the uniporter involved in calcium influx [28,29,30]. This event is considered “non-genomic” since the target is a location, the mitochondrial matrix, vs. a protein, receptor or gene. However, this effect increases cyclic adenosine monophosphate (cAMP) (2nd messenger), likely through activation of adenylate cyclase. The production of cAMP transitions the non-genomic event into a genomic event and induction of expression of a host of genes, both up-regulated and down-regulated. In particular, the DNP → cAMP → cAMP response element binding (CREB) → Brain-derived neurotrophic factor (BDNF) cascade lends itself to the possibility of increased cognition [30,31,32]. Calcium graph was adapted from Liu, et al. 2015 [30].

Modulating the membrane potential by mitochondrial uncouplers has an initial effect based purely on physics with the release of the proton (H^+^), but this effect cascades into cellular remodeling that appears anti-aging [25,26,27]. Since most diseases have a pleiotropic effect, a drug that also has a pleiotropic effect *pro-survival* may be useful in fighting “fire-with-fire” (Figure 1).

## 3. ROS

**Figure 2:**
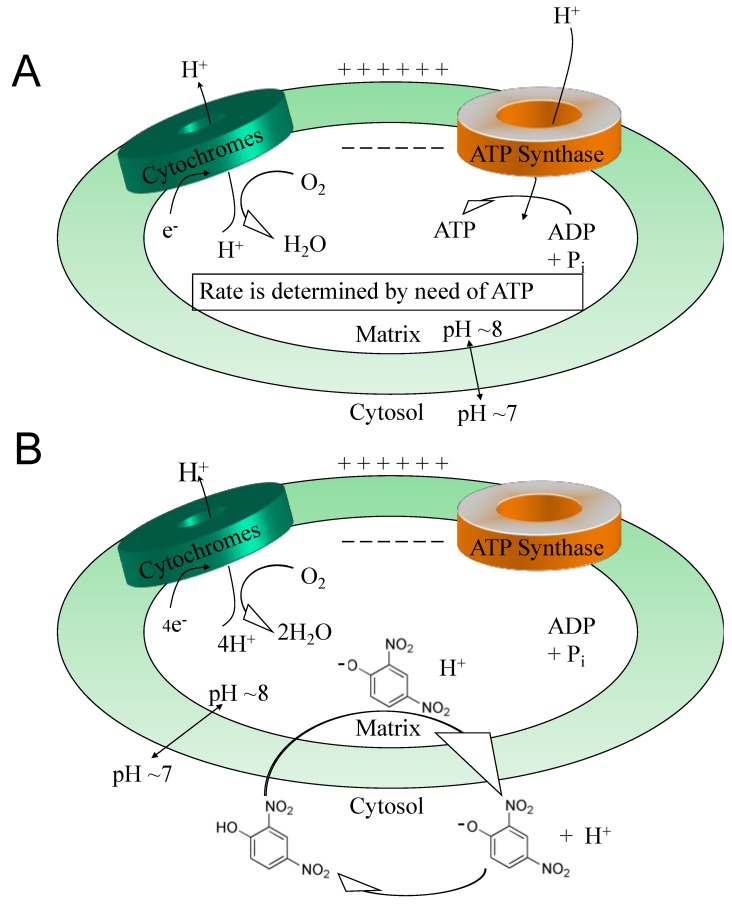
Coupling vs. uncoupling. Maintaining the proton gradient across the mitochondrial matrix and cytosol involves the pumping of protons out of the matrix via cytochromes I, III and IV. (**A**) The coupling of a proton (hydrogen or H^+^) transfer to the synthesis of ATP is a result of H^+^ returning through ATP synthase, causing rotation and subsequent phosphorylation of ADP, thereby yielding an ATP molecule. (**B**) This mechanism is circumvented in the case of chemical uncoupling (i.e., entry of protons without phosphorylation to produce ATP). The proton transfer into the matrix is on a carrier, a weak acid molecule (i.e., 2,4-dinitrophenol) with a unique dissociable proton (H^+^) due to the two NO_2_ “electron withdrawing” groups. Outside of the mitochondria in the acidic environment of the cytosol, DNP is in the protonated neutral form, but attracted to the basic environment of the matrix. Upon entering the matrix, DNP releases the dissociable proton (H^+^). Now in the negatively charged anionic form, DNP is attracted to the acidic environment of the cytosolic space, therefore returning to the cytosol to become reprotonated and the cycle starts over again. All mitochondrial systems remain functional but are accelerated. Reprinted by permission from Springer, Diabetologia (2011) 54:237–244, *Targeting Energy Expenditure* via *Fuel Switching and Beyond*, J.G. Geisler [2].

While antioxidants attempt to neutralize ROSs after they have been made [33,34], uncouplers actually *prevent* overt ROS production [35,36], a potentially more effective point of intervention. This idea was comprehensively reviewed by Dr Alicia Kowaltowski, de Souza-Pinto, N.C., Castilho, R.F. and A.E. Vercesi (2009) [36], who discussed the collective benefits of enhancing respiratory rates by mild uncoupling, as follows: (1) increasing O_2_ consumption prevents formation of superoxide radical anions (O_2_^-^) by decreasing O_2_ tension in the microenvironment, (2) it favors more oxidized levels of respiratory chain intermediates, such as in Complex I and III, known as a substantial source of ROSs, (3) uncoupling keeps NADH levels lower, which prevents ROS formation by mitochondrial matrix flavoproteins, and, (4) it lowers mitochondrial membrane potential (ΔΨ), a condition that thermodynamically disfavors the reverse flow from Complex II to I, thereby decreasing ROS formation. Mitochondrial chemical uncoupling mimics a naturally occurring phenomenon termed, “proton leak” [6,37]. Protons that could be utilized for useful work (oxidative phosphorylation), or the “coupling” of the electronic event into the synthesis of ATP, instead leak across the mitochondrial membrane and are lost as heat. In fact, ~25% of the body’s basal energy is lost by this mechanism and is thought to slow aging by lowering ROS production. Chemical uncouplers work by having the properties of being a weak acid with specifically a dissociable proton (H^+^), that travels the only organelle that has a pH basic environment, the mitochondrial matrix (Figure 2). In an acidic environment, DNP is in a protonated neutral form but is a carrier of a proton, hydrogen (H^+^), and possess the property of being able to dissociate and release its proton upon entering the pH basic environment of the matrix [2]. Upon exiting the matrix, now in its negatively charged anionic form, DNP gets reprotonated back into neutral form in the acidic environment of the cytosolic space of the mitochondria, whereupon it returns back to the matrix to deliver another proton (H^+^). Since oxidative phosphorylation requires an H^+^ returning back into the matrix, but only through the ATP synthase membrane channel to synthesize ATP (coupled), then the process of a proton bypassing this channel is considered “uncoupled”. This change in the proton gradient (electron motive force) causes a momentary drop in the potential causing compensation with an increase in all processes related to the electron transport system in the forward direction. The membrane potential never gets completely back into balance or re-established while a chemical uncoupler is present. It is essential to note, that mitochondrial chemical uncouplers are not inhibitors of the electron transport system, but enhancers [2]. The system must remain completely functional for energy expenditure to be maintained without inhibition to any of the complexes or to ATP synthase. It was established as early as 1933 that the mitochondrial source of energy comes specifically from the oxidation of lipids and sugars, but not catabolism of proteins [38]. It was not established until 1961 by Nobel Laureate, Dr. Peter Mitchell, how mitochondrial uncouplers modify oxidative phosphorylation [39].

Building on Miwa, S. and M. D. Brand (2003) findings in isolated Drosophila mitochondria that the physiology of ROS production is very sensitive to chemical uncouplers by slightly lowering the membrane potential [35], Caldeira da Silva, C., Cerqueira, F., Barbosa, L., Medeiros, M., and A. Kowaltowski (2008), exercised the idea in vivo by putting DNP into the drinking water of a colony of female Swiss Webster albino mice to see what happens. DNP was provided starting at 18-weeks of age until their natural death (>500 days on drug), at a dose ~100 μg/day (human equivalent dose (HED) of 0.45 mg/day) or roughly ~600 times lower than the common daily dose for obesity in the 1930s at ~300 mg/day [40]. The consequence was that the mice on DNP lived *longer* than water alone (placebo). A subgroup after 1 or 5 months of DNP treatment was used to possibly reveal the reasons for increased longevity. These mice had significantly lower circulating triglycerides, glucose and insulin, and less oxidized proteins and DNA in the brain, liver and muscle due to lower hydrogen peroxide (H_2_O_2_) production. The salient point is, that a little mitochondrial uncoupling under chronic regimens, may have a large effect on wellness and even slow ageing.

## 4. Calcium Overload

**Figure 3:**
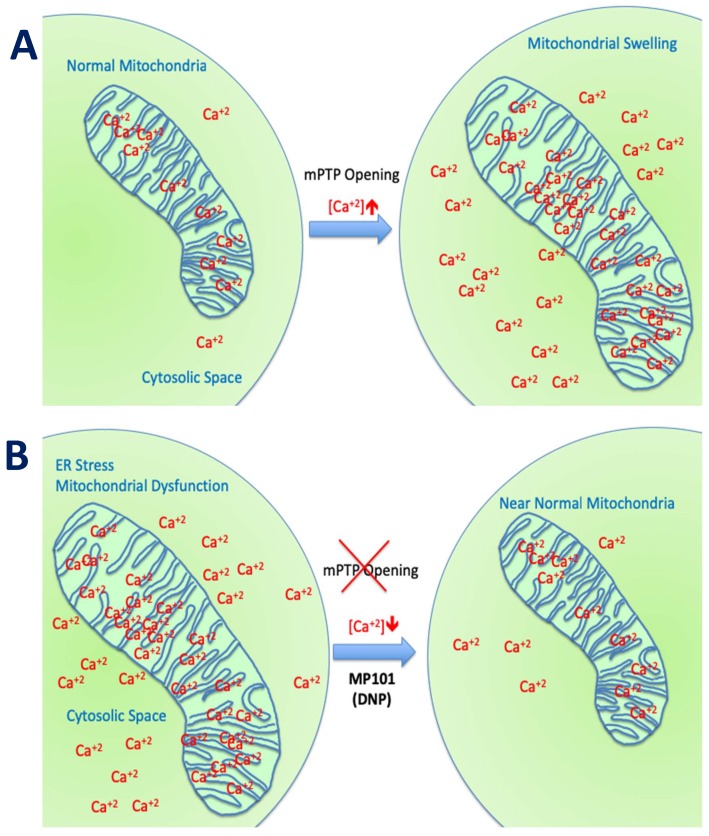
Calcium overload and DNP. (**A**) The cytosolic Ca^2+^ under normal conditions is kept low and released from the mitochondria for signaling [1]. However, for conditions related to unfolded protein response (UPR) that lead to ER stress such as Huntington Disease [41,42] or mutations directly of ER function such as Wolfram Syndrome [43] or loss of dystrophin in the case of Duchenne Muscular Dystrophy [44], calcium levels rise in the cytosol and subsequently in the mitochondria. If the threshold for mitochondrial Ca^2+^ storage is exceeded, the mitochondrial permeability transition pore (mPTP) is formed and the mitochondria are destroyed setting up a cascade for neighboring mitochondrial destruction [16]. (**B**) Calcium overload of the mitochondria may be reduced, even in the presence of a sudden rise of cytosol Ca^2+^ from the ER, in TBI and other conditions of metabolic stress, in the presence of DNP by closing the calcium uniporter channel. Reduced intra-mitochondrial calcium removes the driving force toward apoptosis, thereby saving the neuron, myotube and other at-risk cell types.

Beyond the ROS component, the channel for Ca^2+^ influx into the mitochondria, the “calcium uniporter” is voltage dependent and also influenced by DNP’s effect on the mitochondrial membrane potential. DNP lowers Ca^2+^ influx. This was demonstrated nicely in a model of traumatic brain injury in Dr Patrick Sullivan’s laboratory where they induced TBI with controlled cortical impactor (CCI) and subsequently provided DNP. Using a cocktail of Ca^2+^ “locking” buffers to trap Ca^2+^, altered Ca^2+^ loads could be a monitor *in situ* from isolated from cortical mitochondria. Calcium influx rose from ~800 μM to 1200 μM 3-h post-injury, but with DNP on board, levels were maintained close to non-injured levels of ~800 μM [29]. The estimated Ca^2+^ levels were ~30% higher in the mitochondria without DNP, driving the opening of the mPTP, and subsequently apoptosis [1,16]. In the presence of DNP, ROSs and Ca^2+^ levels were reduced resulting in anatomic brain tissue sparing of the cortical injured area, and even protecting cognition, as determined by a novel object recognition test [29]. This demonstrates the potent neuroprotective early-immediate effects of just one dose of DNP (Figure 3), just by lowering the mitochondrial membrane potential.

## 5. ATP Pool

Mitochondrial uncouplers by definition disrupt the proton gradient that could be used to do “useful work” by the individual protons traveling through ATP synthase to make ATP, but are instead leaked and “wasted” as heat [2]. This effect lowers the membrane potential, generates heat, and increases the availability of ADP. The relative increase in ADP, induces the mitochondria to step-up the rate of energy utilization, through increasing the metabolism of both glucose via glycolysis and lipids via β-oxidation. The augmented NADH and FADH_2_ products, through the electron transport chain, attempt to regenerate the proton gradient and re-establish the potential to drive further ATP synthesis.

Although a lower ATP pool could be detrimental in the short term, it is noteworthy, at 3-days of dosing rats with DNP at 16 mg/kg (HED of 150 mg/day) there was no measurable change in intrahepatic ATP content (ATP pool), as assessed by ^31^P magnetic resonance spectroscopy [45]. It is known that for some diseases, ATP production is low and ROS production is high, which is usually associated with the lower membrane potentials found in dysfunctional mitochondria [44,46,47]. Since DNP lowers the membrane potential, there is a theory that it might selectively push those already marginally functioning mitochondria into mitophagy to shift the global mitochondrial population towards better functioning high ATP producers and lower ROS producers [48]. Additionally, it was shown that 3-days of mitochondrial uncoupling, induced by low concentrations of dinitrophenol (10 and 50 μM) in cultured human HepG2 cells, that there is an adaptive effect in that DNP towards oxidative metabolism, with an upregulation of COXIV and ANT3 gene expression, two nuclear genes that encode mitochondrial proteins involved in oxidative phosphorylation [49]. Glucose consumption, lactate and pyruvate production and growth rate were unaffected, indicating that metabolic adaptation of HepG2 cells undergoing chronic respiratory uncoupling allows continuous and efficient mitochondrial ATP production without the need to increase glycolytic activity. So collectively, it is possible that the net outcome is a higher ATP pool through more efficient mitochondrial functioning under chronic DNP treatment, than could previously be generated without the uncoupler.

## 6. Cognition, Cellular Remodeling and BDNF Induction

Adenylate cyclase activation is partly regulated by changes in Ca^2+^ and magnesium balance [29,50]. It was shown in Dr Sergio Ferreira’s lab (2007) in Brazil that DNP induces cAMP production in hippocampal neurons [51]. As a second messenger, cAMP is known to up-regulate and down-regulate a host of genes. For instance, in rat primary hippocampal neurons cultured with DNP and assayed using a rat 5K DNA microarray chip, 275 genes up-regulated and 231 down-regulated for expression [27]. A number of the pathways that were modulated related to cAMP, adenylate cyclase and dopamine signaling, which are involved in memory, learning and cell growth. *Fkhr,* *Lsamp* and *Pp2c* were up-regulated, and are directly involved in synaptic plasticity.

In a study conducted in Dr Mattson’s lab. (2015), C57BL/6 mice were dosed with DNP at 5 mg/kg and then monitored for gene expression. It was found that CREB (cAMP-response element-binding protein) and BDNF (Brain-Derived Neurotrophic Factor) were upregulated in the cerebral cortex, both factors involved in synaptic plasticity and adaptive cellular responses to stress [30]. Beyond the gene expression changes, it was demonstrated that DNP treatment in wildtype mice improved cognition in the latency model (passive avoidance test). In follow-up study, Geisler, J.G., Marosi, K., Halpern, J., and M. P. Mattson (2017), dosed 6-month old aged C57bl/6 mice for 7-days at 0.5, 1, 5 and 10 mg/kg (HED 2–45 mg/day) and then the cortex was measured for changes in BDNF expression. Interestingly, BDNF was induced ~2.5× in the cerebral cortex at 1 mg/kg, but in a hormetic-like fashion, in that, the peak dropped going from 5 to 10 mg/kg, suggesting “less is more” [52]. The expression changes in cAMP and CREB in the brain, are precisely the pattern required for learning and memory from the work of Dr Eric Kandel [32]. BDNF is transcribed by CREB and BDNF feeds forward its own expression [53]. BDNF is considered to be a highly valuable factor, critical for both neuronal growth and the repair of damaged neurons [53,54,55]. Lower levels are considered to reflect a loss of cognition and several disorders are related to lower levels in the brain, so re-establishing near normal levels could be important to slow or reverse disease progression [56,57,58,59,60,61,62,63]. Interesting and less well known, BDNF is also a myokine outside of the brain, important for muscle biology and strength, and also appears to have anti-diabetic qualities [64,65,66,67,68,69,70,71,72,73,74]. Given the hormetic-like dose effect of DNP on induction of BDNF, such that higher doses, less is made, speaks to the idea of “optimal pharmacology”. Pushing the dose could be counterproductive to the goals of improving cognition, slowing aging and attenuating neurodegeneration. In addition, similar to the idea that 5-buffs of serotonin are different than 1-buff in isolated neurons of Aplysia snails in building long term memory, one dose of DNP is adequate for immediate changes in ROS production and Ca^2+^ overload, but chronic treatment is likely required to see the full pharmacology by establishing steady-state increases of BDNF and other factors to improve long term cognition [30,31,52].

## 7. DNP as Medicine

Before discussing DNP’s applications today as a possible treatment in humans with insidious neurodegenerative diseases, it is crucial to start from the beginning to discuss what happened in the 1930s in humans to put the future in proper context. Historically, DNP was used ~80-years ago in clinical studies conducted at Stanford University to determine the merits of DNP as a weight loss agent in obese patients. DNP was an ingredient in ammunition and it was learned by Dr Tainter, Dr Cutting and Dr Stockton that when factory workers or soldiers were exposed to high doses of DNP orally or inhaled, that they subsequently lost weight [75]. This initial observation of DNP-associated weight loss interested many to explore the compound as a treatment for obesity [38,75,76,77]. With an overwhelming appeal, within one year of publishing the first clinical study with DNP in 1933, well over 100,000 people had already taken this unapproved drug, of unknown purity or impurities or toxicities as a weight loss agent [78]. It appeared that many consumers of the drug were taking it without a prescription or knowledge on how much or monitoring at all. The allure for weight loss was enormous. Sales and distribution came from both physicians and entrepreneurs without a medical background. Clinical studies established a relationship between the dose of DNP and the speed of weight loss. For example, 100 mg/day of DNP resulted in a weight loss of ~1 lb/week, while higher doses of DNP resulted in larger weekly weight losses. An odd doctrine was established early to increase the dosage until ~3 lbs per week (~100 mg t.i.d. or ~300 mg/day) was achieved, viewed as the “optimal” steady-state dose. Toxicities, such as rashes (~7%) and cataracts (0.1%–1%), started to appear at higher DNP doses [78,79,80]. The rashes appeared to go away with discontinuation of DNP, however, in most cases, cataracts required surgery to replace the lens [80]. Others sought even more weight loss and deaths started to appear [81,82]. Since DNP was previously used for industrial purposes as a pigment (yellow) for fabrics, leathers and other uses, it was not considered to be on the black market *per se,* because there were no laws governing the distribution of such a molecule in the early 1930s [83]. In 1938 the Food, Drug and Cosmetic Act was established and finally, distribution between state lines was shut down and DNP was illegal to purchase [83]. It is unfortunate that the standards of medical care in the 1930s did not need to know the mechanism of action (MOA) to provide a drug to patients, as it wasn’t until ~3-decades later (1961) when Nobel Laureate, Dr Peter Mitchell established how this platform works by changing oxidative phosphorylation [39,84]. DNP is used today on the black market, but primarily by weight lifters seeking to remove fat between muscle groups to better define each muscle (termed “cuts”) just prior to a competition. These driven individuals have seen a number of deaths by taking high doses in short periods of time to clear the last bits of fat away [81]. Although these high doses are effective for reducing fat deposits, they are not likely effective for neuroprotection or cognition due to the hormetic-like beneficial effects seen at very low doses, that are likely lost at high doses [52].

**Figure 4:**
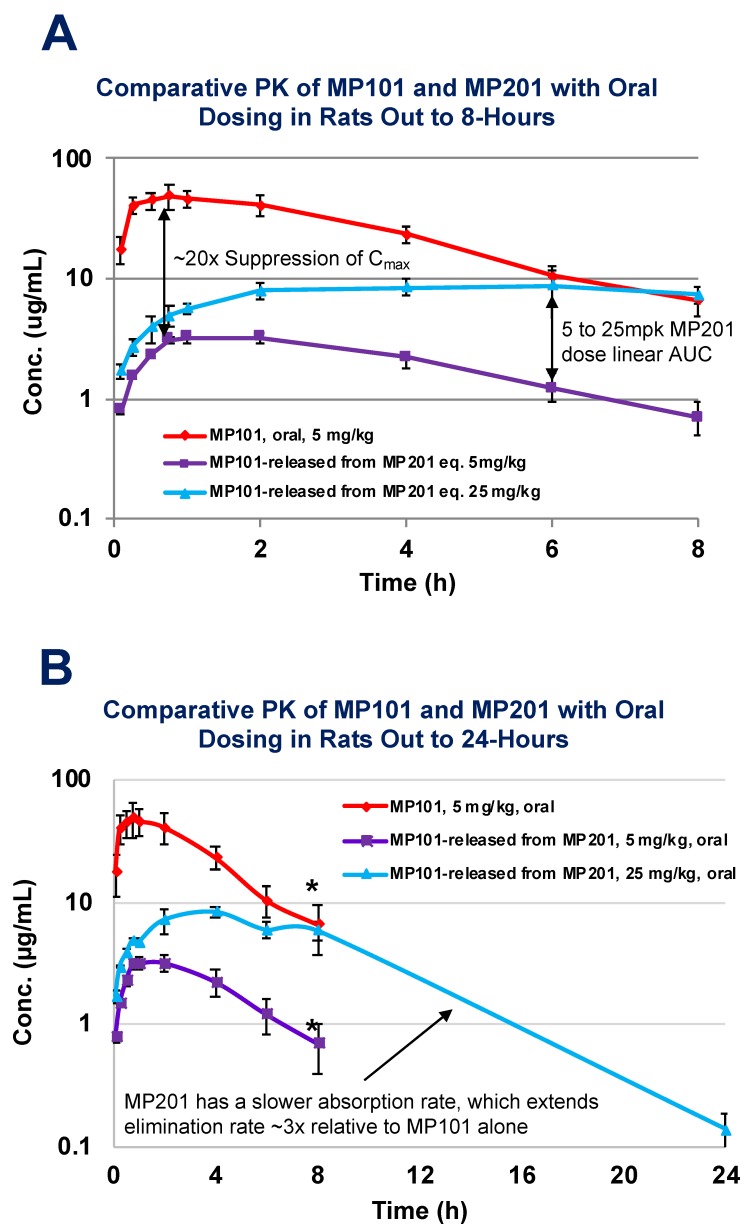
Comparative Oral PK in Rats of MP101 (DNP) vs. MP201 (prodrug of DNP). Male Sprague-Dawley rats (weighing 250–300 g) were housed three per cage with ad libitum access to food and water in the Division of Laboratory Animal Resources, UAMS. Femoral vein-catheterized rats, were treated with MP201 at a single oral dose (N = 4/dose) of 8, 40 and 80 mg/kg (equivalent to 5, 25 and 50 mg/kg of MP101, respectively due to the extra molecular weight) or DNP at 5 mg/kg. Blood samples (0.15 mL) were collected at 0, 5, 15, 30, 45, 60, 120, 240 and 480 min. Plasma samples were prepared and analysis was run using LC/MS/MS spectrometry. (**A**) MP101 (DNP) at 5 mg/kg shows a quick rise and a high C_max_ relative the same equivalent dose at 5 mg/kg of MP201 (adjusting for additional molecular weight). Data shown is MP201’s release of DNP upon cleavage of prodrug linker to an active form (MP101/DNP) with a ~20× suppression of C_max_ compared to MP101. In addition, MP201 has a dose linear AUC going from 5 to 25 mg/kg. (**B**) In compensation for the lower C_max_ relative to MP101, MP201 has a much longer elimination phase (~3×) extending the AUC significantly for a “trickle-like” effect delivering DNP (unpublished). *Below the limits of detection at 24-h time-point of 10 ng/mL.

Considering this baggage from the past and risks, it is often asked why not find a *new* molecule vs. working with DNP? The primary benefit of starting with DNP as a medicine in humans is that the toxicities are largely known and can be monitored. Starting with a new chemical uncoupler throws away the wealth of data in humans (>100,000 people) as all new molecules have their own unknown toxicological risks in humans. The doses where various toxicities have been encountered is already partly known from both studies in humans and non-human animal work [78,80,81]. It is also predicted from studies in a host of neurodegenerative models, that the therapeutic window for desired neuroprotective effects is far below those doses where toxicity is first encountered [28,29,52,85,86,87,88]. The other benefit of DNP is that it is a weak uncoupler, relative to other uncouplers (i.e., FCCP, CCCP, etc.), therefore the therapeutic window is much wider [89]. Strong uncouplers can be used in preclinical models to demonstrate the general effects of this unique platform’s pharmacology, but the latter is unsuitable for humans as a therapy. With that said, DNP does have some room for improvement. Some drugs are modified to start off inactive, and later become active in the bloodstream. These “pro-drugs”, are normally used to help get a drug into the blood that has poor intestinal absorption [90]. This is not the case with DNP, as it has rapid absorption. DNP is likely a Class I molecule similar to ketamine in that no transporters are required for drug uptake [91]. Similar to the goals of lisdexamfetamine dimesylate (Vyvanse), a prodrug of *d*-amphetamine, provided for Attention Deficient/Hyperactivity Disorder (ADHD) such that the child can be dosed in the morning prior to going to school, but remains active long enough to make it home from school for the next dose, we (Mitochon Pharmaceutical Inc.; the author is a founder and shareholder) wanted to create a longer acting DNP [92]. By adding a linker onto the hydroxyl group to displace the hydrogen, DNP becomes inactive and no longer functions as a mitochondrial uncoupler. We were interested to know if making DNP into a prodrug would slow absorption, extend residency time and improve upon the safety index as well as be a new discovery with intellectual property (IP) rights [28,85]. Therefore, Mitochon sponsored work at the laboratory of Dr Peter Crooks (University of Arkansas, UAMS), to develop a series of DNP prodrugs. One of the versions, named MP201, was compared head-to-head with DNP (MP101) in rat pharmacokinetic studies (Figure 4). Studies conducted by Dr Narsimha Penthala and Dr. Zaineb Albayati revealed that with a carbon linker added onto DNP, absorption and conversion of MP201 into the parent molecule (DNP) is considerably slower. Because of this trick, the C_max_ became ~20× lower when comparing 5 mg/kg of DNP (MP101) to equivalent 5 mg/kg MP201 adjusting for additional molecular mass (Figure 4A). In addition, this extended the area under the curve (AUC) ~10× and residency time ~3×, to now allow DNP to be above the limits of detection at 24-h (Figure 4B). Without the prodrug linker, the parent molecule could only be detected out to 8-h (Figure 4A). However, given the pharmacology that is sought with DNP, such as lowering ROSs and Ca^2+^ influx, a longer residency time is desired because it provides a “trickle-like” effect on delivery for near 24/7 neuroprotection. In fact, there appears to be an improvement in efficacy, which likely comes from slow absorption with long AUCs, and lowered toxicities from a lower C_max_, that possibly provides near steady-state redox protection and induction of BDNF for repair.

**Figure 5:**
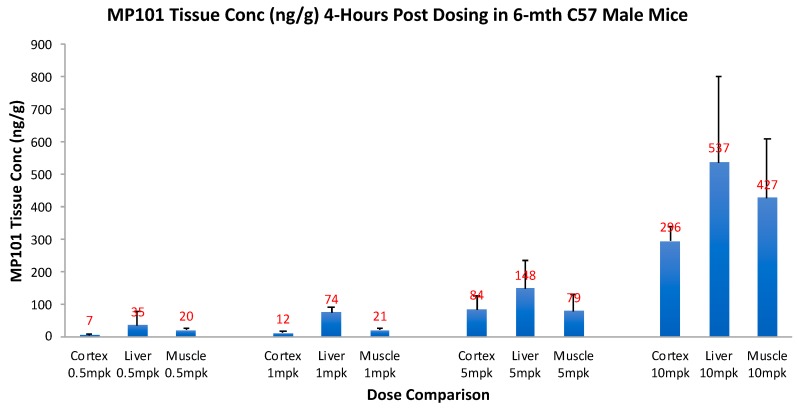
MP101 (DNP) tissue concentrations (ng/g) in mice. Six-month aged male C57/bl6 mice were fasted for 4-h and then provided MP101 at a dose of 0.5, 1, 5 and 10 mg/kg (Mitochon sponsored study conducted at Melior Discovery (Exton, PA, USA)). Four hours later, the cortex, liver and skeletal muscles were harvested to determine tissue penetration levels by LC/MS at Keystone Bioanalytical (North Wales, PA, USA). The brain levels were generally slightly lower than muscle, which was less than the liver (unpublished).

Critical for a drug to be useful as a treatment in neurodegenerative disorders, it is important to know, that not only does it cross the blood-brain barrier (BBB), but that it has good tissue penetration within the dose range intended for efficacy. Therefore, prior to investing in proof-of-concept (POC) studies in neurodegenerative models, we invested in a tissue distribution study. Aged (6 months) male C57/BL6 mice were given an oral dose of 0.5, 1, 5 and 10 mg/kg, and then 4-h later, drug concentrations were determined in the brain cortex, relative to liver and muscle (Figure 5). By simple oral dosing it was determined that DNP gets into the brain cortex with similar concentrations to the peripheral organs (skeletal muscle and liver), but in a descending concentration: liver, then muscle, and brain cortex lowest respectively (unpublished). This range was chosen to determine a reasonable window of the efficacy dose range, but it was not determined until later to be true. This range of 0.5, 1, 5 and 10 mg/kg, correlates with HED range of ~2–45 mg/day of DNP in humans. With this new knowledge, the tissue concentrations in the cortex, 0.5–5 mg/kg appeared dose proportional going from 7 to 84 ng/g, vs. 10 mg/kg at 296 ng/g, so we chose to begin with doses of 0.5, 1 and 5 mg/kg for our initial POC studies. This tissue distribution study confirmed that DNP is orally absorbed across the intestine into the blood, distributes well to first pass liver, slightly less to peripheral muscle, and still has excellent penetration across the BBB into the brain cortex, to potentially be useful against neurodegenerative disease.

## 8. Neuroprotection

DNP may have a broad neuroprotective effect for diseases of known and unknown causes and paradoxically at weight neutral to weight sparing low doses. The pharmacology capitalizes on the idea of pleiotropic effects that create cellular resiliency by changing ROS production, Ca^2+^ handling, mitophagy with mitohormesis, induction of BDNF and cellular remodeling [30,35,40,52].

## 9. Huntington Disease

Huntington Disease (HD) is an inherited adult-onset (~40) neurodegenerative disorder resulting in choreiform stereotypic movements, loss of muscle coordination, speech, psychiatric manifestations, eventual dementia and premature death [93]. HD has a known genetic mutation in the huntingtin gene, a “tri-nucleotide repeat expansion disorder” of CAG (glutamine) [94].

As far as the author knows, no studies were ever conducted with DNP in compromised animal models with a germline mutation prior to the study in 2015 conducted at Johns Hopkins in Dr Wenzhen Duan laboratory [86]. All prior studies cited in the literature with DNP were in wildtype animals, which are considerably stronger and healthier. The N171-82Q is a knock-in of N-terminal fragment of huntingtin protein (Htt) with 82 CAG repeats for glutamines [86]. So, it was completely unknown if DNP would be harmful or neuroprotective in such a fragile disease model. Here, DNP was provided to a mouse model of Huntington Disease (N171-82Q) for over 17-weeks (119 days) of chronic oral once-per-day daily dosing in a full dose response of 0.5, 1 and 5 mg/kg with a benchmark control in a wildtype cohort treated at the highest dose (5 mg/kg).

The original characterization of N171-82Q showed a number of developmental and behavioral abnormalities, including loss of coordination, tremors, hypokinesis and abnormal gait, before dying prematurely, but did not suggest oxidative stress as a mediator of neuronal damage as thought to occur in humans with HD [95,96,97]. However, after 17-weeks (26-weeks of age), there was a significant increase of isoprostanes in the cortex of placebo HD mice, but the 1 mg/kg DNP treated HD mice (only dose tested), brain isoprostane levels were equivalent to wildtype [86]. Most important was that both the spiny neurons (particularly lost in HD) and general neurons in 1 mg/kg DNP treated HD mice also appeared equivalent to wildtype brains, explaining the preservation of brain volume determined by MRI, and preserved motor skills on a rotarod or balance/tapered beam. The latter is important as choreiform forced dance-like abnormal movements is quite severe in HD patients [98]. This study was the first demonstration that DNP was neuroprotective in a germline disease model. It was also the first demonstration of the concept that DNP has a hormetic effect, where the highest dose (5 mg/kg) lost its effect from 22 weeks to 26-weeks of age, but the two lower doses (0.5 and 1 mg/kg) still had a striking effect for preserving behavior on the balance beam. This phenomenon will be seen to be repeated in other models and suggests that low doses that modulate the mitochondria’s physiology are more powerful in neuroprotection than higher doses. The 1 mg/kg DNP mouse dose is equivalent to ~5 mg/day for an HD patient or ~60× lower dose in comparison to the 1930s of ~300 mg/day for obesity in humans. A dose even lower than 0.5 mg/kg (HED ~2 mg) may also be effective as this dose appeared equivalent to 1 mg/kg on behavior, but was not evaluated for spiny or general neuron levels by Western blot [86].

## 10. Alzheimer’s Disease

Different from a rare and middle-aged onset with a known mutation like Huntington’s disease, Alzheimer’s disease (AD) is a neurodegenerative disorder that manifests itself most often in the elderly. Except in rare cases, AD has no known genetic cause and representing ~73% of all dementia [99]. The hallmark of AD is the loss of short-term memory. Similar to many neurodegenerative diseases, however, onset is usually gradual and of unknown origin in the 2nd half of life and thought perhaps to be due to multifactorial causes, including sequestering of mutations over time, simultaneously with a decline in repair, to tip the scales leading to mitochondrial dysfunction [100]. Perhaps the possible lack of success in so many clinical trials is in part due to a focus on targeting downstream events (plaques and tangles), when the issue is upstream at the mitochondria [101]. In support of this idea, are the findings that isoprostanes (biomarker of ROSs) were significantly elevated in an aged mouse model of AD relative to wildtype littermates 4-months *prior* to the first visualization of plaques [102]. The data suggest that mitochondrial stress (isoprostanes) precedes plaques and AD patients also have much higher levels of mitochondrial stress than non-AD patients [21]. Interestingly, it appears that DNP also has direct anti-amyloidosis properties in preventing Aβ fibrillogenesis and disaggregation of previously formed Aβ_1-42_ aggregates. In vitro experiments by De Felice, F.G., Houzel, J.C., Garcia-Abreu, J., Louzada, P.R., Jr., Afonso, R.C., Meirelles, M.N., Lent, R., Neto, V.M., and S.T. Ferreira (2001) showed that DNP in a dish can block formation or reverse aggregation of Aβ_1-42_ fibrils directly, and in the presence of hippocampal neurons, can prevent Aβ_1-42_ cytotoxic effects and subsequent cell death [103]. These findings were followed up in rats in a toxin-induced in vivo model of AD, where Aβ_1-42_ was directly injected into the left hippocampus alone, or into the right hippocampus combined with DNP, and left to age a week. Follow-up 8-days later revealed the striking findings of plaques on the left side and almost no plaques on the right side, or ~86% reduction. It would be interesting to see if this effect could be seen with systemic delivery after plaques are already formed to follow-up on the in vitro work for late-stage AD, and in a model where plaques naturally form along with the rise of isoprostanes to show both can be prevented, representing early stages of AD.

Mitochon Pharmaceutical sponsored an AD animal study with DNP under chronic treatment in Dr Mark Mattson’s laboratory at the National Institute on Aging (NIA). Specifically, APP/PS1 double mutant mice, a model of AD, were dosed with DNP once-per-day orally at 0.5, 1 and 5 mg/kg for 4-mths (120 days) [52]. At the end of 4-months, the mice were evaluated for spatial learning and memory tasks in the Morris Water Maze where a hidden platform was submerged in one quadrant. Over the 7-days of training, it was only the lowest dose of DNP (0.5 mg/kg) that protected the ability to learn quickly. When the hidden platform was removed on the 7th day, the 0.5 mg/kg mice retained their memory of the platform location for 3-days by precisely traveling over to where the hidden platform was and crisscrossing. This demonstrated the striking effect on preservation of short-term memory at very low doses of DNP relative to placebo. Curiously, the higher doses (1 and 5 mg/kg) had no effect, again suggesting a hormetic-like effect. The 0.5 mg/kg dose of DNP in mice is theoretically equivalent to ~2 mg/day of DNP in AD patients and expected to be weight neutral (>100× lower than 1930s). The data suggest that low doses of DNP over an extended period of time can have a powerful effect on Alzheimer’s onset, and perhaps even general aging. It parallels the study where lifespan was increased in wildtype mice treated approximately their entire life at only ~100 μg/day with DNP [40]. Follow-up work is required to elucidate the mechanism of why the lowest dose had such a striking effect, but not the higher doses. Possibly even lower doses than 0.5 mg/kg could have an effect. Follow-up studies could monitor plaques and isoprostanes over time with chronic DNP treatment. Further, the pharmacology may be improved with a prodrug of DNP with longer residence time [28,85].

## 11. Parkinson Disease

Parkinson disease (PD) is a progressive neurodegenerative disorder characterized by a loss of motor function, including muscle rigidity, resting tremor, bradykinesia and postural difficulty. Parkinson Disease is associated with mitochondrial dysfunction, but etiology of the neurodegeneration is unknown [104], though the abnormal accumulation of alpha-synuclein may be responsible [105]. Mitochondrial impairment, free radical-induced injury and inflammatory mechanisms have been postulated to play a role in the neurodegenerative processes of PD.

Sirt3 KO mice appear to have increased striatal vulnerability [42]. In a Mitochon Pharmaceutical sponsored POC study conducted by Joshua Halpern in the laboratory of Dr Mark Mattson’s laboratory at the NIA, Sirt3 KO mice were injected with 6′OHDA in the right striatum and the following day started a 2-week treatment of oral DNP at 1 and 5 mg/kg (unpublished). The idea was that over the course of two weeks, 6′OHDA will specifically be fatally toxic to the dopamine-producing neurons and simultaneously, the pharmacology of DNP would potentially protect the neurons [106]. After two weeks of dosing, the animals were sacrificed for tyrosine hydroxylase (TH+) neuronal cell counts, revealing that DNP treated mice had significantly attenuated the loss of dopaminergic neurons. In a similar acute model of PD, DNP was administered for 12-days at 1 and 5 mg/kg prior to receiving a 20 mg/kg i.p. injection of 1-methyl-4-phenyl-1/2/3/6-tetrahydropyridine (MPTP) four times to induce PD motor dysfunction [9]. DNP attenuated both the motor dysfunction and loss of dopaminergic neurons. These studies represent early-stage Parkinson Disease. Further, if 6′OHDA is injected into medial forebrain bundle (MFB) instead of the striatum, then both depots of dopamine, the ventral tegmental area (VTA) and substantia nigra (SN) show loss of all dopaminergic neurons, representing a more aggressive and late-stage Parkinson model [107]. In a Michael J. Fox Foundation sponsored study, on behalf of Mitochon, rats were injected with 6′OHDA into the MFB and waiting for 10-days prior to intervention with DNP. The consequence was a ~99% loss of dopamine neurons and no protective effect at this very late post-treatment date (unpublished). Since it is likely that all the TH+ neurons were already dead at this point, the study confirms the idea that the best intervention time is early after diagnosis.

Recently, Dr Yuki Kishimoto in Dr Mark Mattson’s laboratory conducted a study representing early Parkinson Disease with 6′OHDA to achieve ~60–70% kill off in wildtype mice of SN pars (manuscript under review). Mice were tested in a head-to-head study with a prodrug of DNP (MP201) vs. MP101 (DNP). Both compounds, at all doses, provided significant behavioral protection preserving grip-strength and motor skill coordination on the rotarod. However, only the lowest dose of MP101 (0.5 mg/kg) had a significant protective effect on TH+ counts, with the lowest dose of the prodrug, MP201 (8 mg/kg), having more than twice the protective effect on a preventing a reduction of TH+ counts compared with 0.5 mg/kg MP101. Similar to AD the study, data suggests that low and sustained doses of DNP, provide optimal pharmacology in PD.

## 12. Multiple Sclerosis

Multiple sclerosis (MS) is an autoimmune disease in which myelin-reactive autoantibody and lymphocytes migrate out of lymph nodes into circulation, crossing the blood–brain barrier (BBB), and aggressively target putative myelin antigens in the central nervous system (CNS), causing inflammation, demyelination, axonal injury, astrogliosis, and ultimately, neurodegeneration [108,109,110,111]. Again, on-set is typically mid-life (~40’s), but life expectancy is near normal, although the quality of life is significantly compromised. There are however some rare forms of MS called acute fulminant or Marburg acute MS with possible death within a few years upon onset [112].

Postmortem comparison of MS brain to non-MS, suggests changes in gene expression related to mitochondrial ATP production specific to neurons and Ca^2+^ mediated demyelination [113]. Using electron microscopy (EM) to look at mitochondrial morphology in the gold standard mouse model of MS, Experimental autoimmune encephalomyelitis (EAE), the number of Y-shaped mitochondria was significantly increased in axons of the EAE spinal cord. Immunohistochemistry revealed dysfunction of mitochondrial fusion/fission machinery in EAE spinal cord axons [114]. A series of studies were conducted in the laboratory of Dr Yoshio Bando at the Asahikawa Medical University, Hokkaido, Japan using DNP (MP101) and MP201 to evaluate the merits of this approach in two independent models of MS, the EAE and cuprizone model (manuscript under review). The EAE model attempts to mimic the human condition where antibodies are raised against the body’s own myelin by injecting wildtype mice with a short peptide of myelin. This peptide then induces an autoimmune response, resulting in paralysis peaking at ~15-days post-immunization. Seven days post-immunization, both compounds were provided for two weeks, followed by 3-weeks with no treatment. Each day, all the mice were scored for degree of paralysis (clinical scores 1–5) for 42-days prior to necropsy. Interestingly, both MP101 and MP201 had a striking effect attenuating paralysis compared with placebo, analyzed by ordinal regression analysis (see Supplementary Movie S1 MP101 and MP201 Prevents Paralysis in EAE Model). Paradoxically, although DNP was historically a weight loss drug, here it statistically preserved body weight at all doses compared to the placebo arm, which showed signs of wasting due to paralysis of hind limbs. Interestingly, even though the mice were off the drug for 3 weeks prior to necropsy, there was still a statistical increase in BDNF protein levels found in the spinal cord. Western blot of the spinal cord also revealed that MBP (Q9) and SMI32 proteins, markers of axonal damage, were absent in the treatment group, but present in the placebo. DNP treatment induced MBP (marker of normal myelin vs. Q9), COX4, CNPase and synaptophysin, relative to placebo. Expression analysis showed a lowering of iNOS, IL-1β, TNF-α and ATF3 in the treatment arm relative to placebo were markedly elevated.

The cuprizone model is completely independent of the immune system [115]. Cuprizone is a chelating agent of copper and when added to the chow, it lowers the essential dietary copper, preventing the formation of myelin sheaths by oligodendrocytes. We performed a DNP treatment with MP101 and MP201 in the cuprizone model, started at the same time that cuprizone was added to the chow (manuscript under review). This continued out for 30-days prior to necropsy. Similar to the EAE model, immunohistochemistry and EM showed that in the corpus callosum of controls, that both the myelin and the axons were destroyed, whereas there was very little effect on demyelination and the axons were intact with MP101 and MP201 treatments shown by electron microscopy.

In progressive MS patients, it was shown that moderate exercise for 30 min stimulates BDNF production [116]. Others have shown that BDNF may be a promising therapeutic for MS [117,118]. So, the idea of treating MS patients with low dose DNP to lower ROS and as well as inducing BDNF to attenuate the next relapse could be a breakthrough therapy, especially for the more aggressive condition of progressive MS.

## 13. Optic Neuritis

Optic neuritis (ON) can be an early sign of MS and sometimes referred to as pre-MS [119]. It is associated with demyelination of the optic nerve and the possible loss of the retinal ganglion cells (RGC) [120]. High contrast vision recovery in most cases is seen without major damage with steroid treatment, but it is often observed that there is a persistent remnant loss of contrast vision sensitivity, motion perception and vision related to reduced quality of life [121]. This loss of sensitivity effects the contrast vision needed to easily read newspapers with grey-on-grey or driving in cloudy weather. Studies Mitochon Pharmaceutical has performed already with Dr Bando in models of MS with DNP and the prodrug of DNP suggested protection from the autoimmune effects in the EAE model of MS. The same EAE model is used to study optic neuritis, but the starting time to treat is later. In collaboration (2017) with Dr Kenneth Shindler’s laboratory at the Scheie Eye Institute at the University of Pennsylvania, we ran a study to determine the possible merits of MP201 for ON [85]. In this case, MP201, a prodrug of DNP was provided once-per-day for two weeks after the EAE mice had progressed into ON, mirroring the human scenario of when a patient would likely come in for treatment with significant pain and rapid vision loss. The results show that treatment had a significant effect on protecting the neuron bodies (RGC), preserving the optic nerve axons from demyelination and kinetics of the eye to focus and track an object called optokinetic response (OKR) [85].

## 14. Diseases of the Threatened Tissue

An additional application for DNP is in patients with illnesses due to body trauma and preventing the acute consequences to the “threatened tissue” or penumbra. Patients with severe burns, polytrauma such as high-speed motor vehicle accidents, falls from a height, traumatic brain injury or concussion, shock, sepsis, ischemic stroke, hearing loss due to explosions or repetitive loud noise, all fall into this unique category with a huge unmet medical need. For example, severe burn victims are typically some of the *most*-sick patients at a hospital, with the risks of sepsis, shock, temperature, fluid and electrolyte shifts, pneumonia, multiple surgical procedures, and pain [122,123]. Trauma from such cases results in a cascade from the directly injured tissue releasing ROSs, that subsequently damage neighboring neurons, myotubes, or other cell types [124,125]. There have been a variety of attempts to lower cellular stress, such as administering anti-oxidants, however, these drugs typically have limited tissue penetration, plus they lower ROSs after they have been formed [33,126]. It has been shown that modulating the mitochondrial membrane potential has a significant impact by preventing ROS formation, which is a more attractive intervention point [35]. Treatment for trauma patients is likely to be relatively short in duration, but the timing of intervention, ideally early to immediate, is likely to be more useful for success to attenuate further damage. Here are some examples:

## 15. Ischemia

A fascinating example of DNP’s capacity to become protective for the threaten tissue or penumbra was elegantly demonstrated in a rat model of cerebral stroke. The common carotid artery (CCA) and middle cerebral artery (MCA) was blocked for 2 h in a rat and then subjected to reperfusion for one additional hour, before administration of DNP with on i.p. injection at 5 mg/kg [87]. This scenario was chosen to replicate the human conditions in how long it might take in an attainable ideal clinical setting for a stroke code to get a person to the hospital, get a CT scan, and receive intravenous tissue plasminogen activator (tPA) to dissolve the clot, generally within ~3-hours. M2 artery was chosen, because it was a plausible infarct to recovery from, whereas M1 is too large and M3 has too limited tissue damage to be able to assess a protective effect. It is also known that the early stages of ischemia are associated with high ROSs. Twenty-four hours after the DNP intervention, the rat brains were evaluated and found to have ~40% reduction in infarct volume relative to placebo. In ischemic stroke, the data suggests that the first line of care would be to provide tPA back-to-back with DNP, or to even administer the DNP prior to tPA so that it is already in the blood as the clot is dissolved, so that as soon as blood flow is restored, DNP is there to reduce ROSs released from the dying cells and protecting neighboring cells. Further, mechanical removal of cerebral intravascular occlusion by interventional neurosurgeons and neuroradiologists has found efficacy up to 6 h after onset of stroke [127]. This extended window of efficacy to 6 h provides a longer window where DNP might provide biophysical protection from ROS formation at the late reperfusion stage.

One dose of DNP had an early immediate biophysical effect on ROS production and Ca^2+^ influx since both are mitochondrial membrane potential modulated. Infarct volume may have been further reduced if treatment continued for more than one dose, potentially over multiple days to weeks.

There may be a role for DNP on the continuum from reducing acute injury as a neuroprotectant drug, to a neurorestorative drug as part of rehabilitation over the year after a stroke, as the remaining brain circuitry attempts to rewire and repurpose, combined with reinforcement from steady production of adult neural stem cells. It is now known that under chronic treatment, DNP increases levels of BDNF in the brain (consider “fertilizer of the brain”). The local bathing of neurons with BDNF may further assist in the repair of damaged neurons and re-establish communication among the fine neurite connections while maintaining ROSs at low levels for optimal recovery.

## 16. Sciatic Nerve Damage (Crush Injury Model)

To demonstrate the effects of DNP to block the decline of function post-nerve injury, da Costa, R.F., Martinez, A.M. and S. T. Ferreira (2010) used a “crush injury” mouse model of the sciatic nerve [88]. Under anesthesia, the sciatic nerve was exposed on the left hindlimb and clamped for 1-min. This induces extensive nerve damage by 48-h. Immediately after the trauma, the skin is sutured closed and subjects are administered one dose of 0.06 mg/kg i.p. injection of DNP. One group received an additional dose 24-h later and a second group 3 more doses, every 12-h, compared to a placebo and sham group. At 48-h later, the mice undergo necropsy and the sciatic nerve is removed, fixed in formalin and sectioned to evaluate morphological changes. Data showed that 48-h post-injury, that the sciatic nerve had extensive edema and decrease in fiber density in the placebo group. However, in the group with 2-doses of DNP, there was a clear reduction of both edema and degeneration, but with 4-doses there was almost no sign of nerve damage and it was difficult to distinguish from the sham control group. In a second cohort, they were allowed to age 6-weeks post-injury, and both the 2 and 4 doses of DNP groups showed partial to complete paw toe extensions, vs. placebo, which did not have a recovery. The study suggests that with the early-immediate treatment of DNP or other uncouplers, that the long-term consequences of nerve trauma may be attenuated, but the timing of treatment is critical. It also demonstrates that even very low doses can have a powerful neuroprotectant effect since 0.06 mg/kg in a mouse is a HED of ~0.3 mg (1000× lower than the 1930s regime for obesity at 300 mg/day).

## 17. Traumatic Brain Injury (TBI)

Currently, there is no FDA approved therapy for TBI or concussion and it is a major health issue for both civilians and the military. It is well known that TBI results in impaired mitochondrial respiration and changes in Ca^2+^ handling that leads to the opening of the mPTP [128,129]. Cyclosporine has been studied as a possible treatment for TBI [130,131]. It is originally described as an immunosuppressant of T-cell activation, but its neuroprotective effects are unrelated to immunosuppression. After TBI in animal models, it is the ligand for and binds mitochondrial cyclophilin D, preventing its participation in and therefore inhibiting the formation of the mitochondrial permeability transition pore (mPTP). Cyclosporine is in human clinical trials for TBI, and other cyclosporine derivatives are under investigation in animal models [132,133]. In animal models of TBI, it has been shown that rapid brain cortical necrosis occurs at the site of impact due neuronal necrosis and apoptosis thought to be caused in part by massive excitotoxic neurotransmitter release, Ca^2+^ overload, overt mitochondrial ROS production and formation of the mPTP inducing the release of toxic mitochondrial proteins into the cytosol, including cytochrome C, caspase activators and apoptosis-inducing factor. Cyclosporine has been shown to be strongly neuroprotective in this model. Challenges for cyclosporine (ring structure with high molecular mass of 1202 g/mol) are its insolubility in water requiring detergents or lipid emulsion excipients, immunosuppression side effect, and poor penetration of the intact BBB, making it suitable only for more severe TBI where the BBB has already been disrupted, and so is not suitable to treat concussions or the more common minor forms of TBI.

Uncouplers as well have been extensively studied in models of TBI. Pandya, J.D., Pauly, J.R., Nukala, V.N., Sebastian, A.H., Day, K.M., Korde, A.S., Maragos, W.F., Hall, E.D., and P. G. Sullivan (2007) showed that both FCCP and DNP provided to a rat post-controlled cortical impactor (CCI), that with just one dose, the effects of trauma can be attenuated to some degree leading to tissue sparing ~12% greater than vehicle and cognitive function in Morris Water Maze (*p* < 0.01) [29]. The work showed that the pharmacology of uncouplers was similar to FCCP and DNP, however, FCCP has a considerably narrow safety index relative to DNP [89]. The acute MOA was shown to lower the mitochondrial membrane potential, abolish overt ROS production, and reduce driving force of uniporter Ca^2+^ influx into the matrix, thereby reducing Ca^2+^ overload that leads to apoptosis of the cell [29,134]. Recently, this approach was followed up, Hubbard, W.B., Harwood, C.L., Geisler, J.G., Vekaria, H.J. and P. G. Sullivan (2018), with the prodrug MP201, which extends DNP residency time. In addition, 2-weeks of chronic treatment was added on to provide the full pharmacology with the induction of BDNF [28]. This may provide the best possible outcome in the brain for additional repair with a neurotrophin bathing the recovering neurons, as well as continuous suppression ROS production that may extend beyond a one dose treatment [29]. Markers of ROSs [4-hydroxy-2-nonenal (HNE) and protein carbonyls] in the cortex was lower in the MP201 mice, compared to the vehicle after 2-weeks of daily chronic oral treatment, as well as a significant improvement in cortical sparing (~38%) and most importantly, cognitive improvements [28]. The large neuroprotective effect of DNP and prodrug in TBI will likely be optimized through further studies, both in terms of dose and length of treatment. DNP and prodrug have the advantage over cyclosporine of being a much smaller molecule (DNP’s molecular mass 184 g/mol), water-soluble, easily formulated as an oral and intravenous agent without the need for complex solubilization systems or toxic excipients, and easily crosses the intact BBB to reach the brain. In addition to potentially becoming a treatment for severe and moderate TBI, DNP and prodrug could be given as a one-dose oral capsule to the over 2 million people presenting to emergency rooms each year in the USA who have a concussion or other mild head injury.

## 18. Hearing Loss

It is established that overt ROSs can be produced due to loud noise and certain ototoxic drugs can damage the hair cells of the ear, resulting in either temporary or permanent hearing loss [135]. Exposures to blast waves and continuous noise not only damages the inner ear but causes cell death in the hippocampus, suppress neurogenesis and impairs memory function. Aging as well can manifest in mitochondrial dysfunction leading to hearing loss [136]. It is emerging that attempts to treat hearing loss by targeting downstream issues has so far not been effective [137]. It may be more fruitful to target oxidative stress upstream at the mitochondria that may be causative for many disorders [19,20,21,102]. In collaboration with Mitochon Pharmaceuticals, a pilot study was conducted in Dr. Richard Salvi’s laboratory (University of Buffalo, NY) using both MP101 and MP201 head-to-head (unpublished). Auditory function was assessed with the compound action potential (CAP) which reflected the summed neural output from the auditory nerve evoked by sound stimulation. In this study, the CAP was evoked by tone bursts of increasing intensity (dB SPL) to measure the CAP input/output functions. CAP input/output functions were measured over a range of frequencies. Figure 5 shows the CAP input/output functions measured at 12, 16, 20 and 24 kHz. Larger CAP amplitudes and lower CAP thresholds are indicative of better auditory function than smaller amplitudes or higher CAP thresholds.

**Figure 6:**
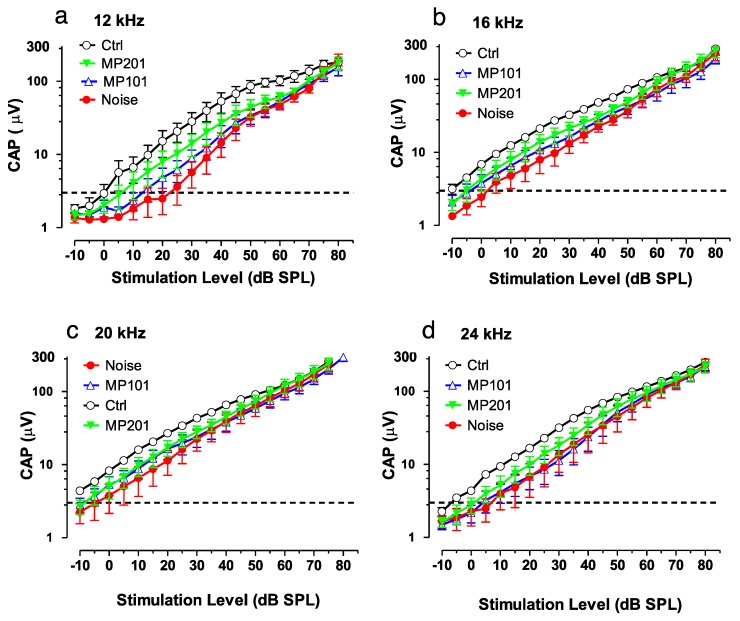
MP101 and MP201 are protective for hearing loss due to noise trauma. The compound action potential (CAP) amplitude is plotted on a logarithmic on the *y*-axis with the intensity of noise (decibels) on the *x*-axis. The CAP input/output functions are shown at (**a**) 12, (**b**) 16, (**c**) 20 and (**d**) 24 kHz. The data indicate that the CAP amplitudes were larger (better) in the noise-exposed groups that received MP201, versus the Noise alone group and statistically significant at 16, 20 and 24 kHz (*p*-value < 0.0001), but not 12 kHz (unpublished). Repeat measures by ANOVA.

The results suggest that MP201 and to a lesser extent MP101 can be useful to preserve auditory function (Figure 6). The pilot study details are as follows: Rats, *n* = 6/group: control, noise alone, noise + MP101 (5 mg/kg), noise + MP201 (80 mg/kg). Noise exposure: 8-h, 105 dB, noise band 8–16 kHz. Treatment duration: 5 days by oral gavage q.d. starting day of noise. Endpoint: Compound action potential (CAP) recorded from the round window membrane 1-week post exposure. CAP Threshold (4 μV) was used to assess otoprotection in this study and reflects the lowest sound intensity (dB SPL) needed to elicit a just detectable response (analogous to the threshold for hearing). As the sound intensity increases above the threshold, the amplitude of the CAP becomes larger. For CAP thresholds, the results show that at 12-kHz, with 80 mg/kg MP201 the CAP threshold was approximately 5 dB SPL, whereas the CAP threshold in the placebo control group (Ctrl) was ~25 dB SPL (sound pressure level). With MP101 treatment, the CAP threshold was ~15 dB SPL, 10 dB lower (better) than placebo, control, but not as effective at MP201 which was 5 dB lower than MP101. Moreover, the CAP amplitudes in the noise-exposed group that received MP201 were consistently larger than the noise alone group; the CAP amplitude in the MP201 group was significantly larger than those in the noise alone group at 16, 20 and 24 kHz (*p*-value < 0.0001), but not at 12 kHz. CAP amplitudes in the noise group treated with MP101 were not significantly different from the noise alone group suggesting that perhaps the longer residency time of the MP201 prodrug provides superior pharmacology to MP101. The larger the CAP amplitudes in the MP201 treated group indicate a stronger neural response to sound than those in the noise alone group. Noise exposures typically produce the most damage at frequencies above the noise exposure. Noise exposure tested here was from 8–16 kHz and we see the most protection (bigger CAP in the MP201, than MP101) at 16, 20 and 24 kHz. The data bodes well with TBI protective effects since hearing loss is often accompanied by head trauma [138].

## 19. Neuromuscular Diseases

With the knowledge that DNP readily passes from the blood into skeletal muscle, that it lowers Ca^2+^ overload and isoprostanes in neurodegenerative models, it was plausible that it may have merit for neuromuscular diseases associated with high isoprostanes and poor handling of calcium. Duchenne Muscular Dystrophy (DMD) and Becker Muscular Dystrophy (BMD) are well known to have elevated isoprostanes [20]. This suggests that ROSs coming from muscle mitochondria are overtly elevated and both DMD/BMD have mitochondrial dysfunction [44]. The loss of Dystrophin on the X-chromosome results in muscle atrophy in young boys. This is an aggressive phenotype, with leg braces at ~8, wheelchair at ~10 and death ~20 due to either loss of heart or diaphragm muscle. Dystrophin is only expressed in two tissues, muscle and brain [139,140,141]. Although muscle loss is largely the focus of therapies, these young boys typically have cognitive impairments as well [142]. There is large heterogeneity, but cognitive impairment can range between mild to severe mental retardation, with impairments in verbal skills, reading, and both short and long-term memory. It is believed that with the loss of dystrophin, the mechanism of muscle loss is largely due to a significant elevation of free cytosolic Ca^2+^ from the sarcoplasmic reticulum (endoplasmic reticulum of myotubes) which destroys the mitochondrial population (lowering ATP availability) and cascades into apoptosis of the myotube [14,143].

The background of genetically modified mice is often critical in the accuracy of reflecting the human scenarios, as in the case of ALS and neuromuscular diseases [144,145]. For many years, the gold standard model for DMD has been the mdx knockout on a C57BL/10 background. However, unlike humans with DMD, the mdx-C57BL/10 muscles get bigger, with no cardiac impairments or replacement of muscle volume with adipose or cycles of degeneration and regeneration of muscle from satellite cells. These mice do show a weakening of diaphragm strength, but not significant inflammation seen in young boys. Recently, the *mdx* mouse model was moved onto a DBA background, that now shows the early inflammatory “storm” and muscle atrophy of both the diaphragm and cardiac tissues seen similar in humans.

In collaboration with Dr Lee Sweeney (University of Florida) and Mitochon Pharmaceuticals, an extensive series of studies were conducted using the mdx mice on a DBA background (manuscript under review). The work was conducted by Dr David Hammer and Zachary Wakefield (graduate student) in *mdx*-dba mice post-weaning. The mice were provided DNP at 1 or 5 mg/kg PO, q.d., N = 10/dose from 4-weeks of age out to 16-weeks, 3-months in total. The primary endpoint was diaphragm strength described earlier [146]. This particular age of the *mdx* model corresponds to very young boys near the premanifest pediatric years, just prior to severe inflammation and muscle atrophy, and into teenage years, resulting in becoming wheelchair-bound [147]. To benchmark DNP’s pharmacology to the current standards of care, a prednisone cohort was added alone or in combination with DNP (1 mg/kg). The combination was added to determine if DNP had any blocking of the effects to prednisone’s pharmacology. The stand-alone group of prednisone was to determine if DNP had merit beyond the standard of care. Glucocorticoids, like prednisone, may have a double-edged sword. In young boys, it dampens inflammation, but in later years accelerates muscle atrophy, suggested that intermediate dosing may provide a better balance [148]. Others report a “lifespan” positive effect [149]. According to the results, DNP treatment produced the ‘highest diaphragm strength out of any other drug tested in this severe mdx model”. Histologically, the drug benefits the diaphragm fiber homogeneity reduces the number of calcifications but does not affect fibrosis. The hearts appeared to have better muscle bundle organization and fewer pockets of inflammation than the control or prednisolone treatments. The DNP mice also appeared more social and active than mice receiving other treatments, suggesting that some positive behavior modification may exist. In follow-up studies from weaning out to 6 months or from 3 months of age to 6 months, the protective effects were lost. This suggests that on translation to humans, the pharmacology may have an optimal effect in early stages, but perhaps not in adults (data not shown). It is possible that with early intervention, DNP treatment could have a meaningful effect for muscle protection during the high growth pediatric period, which could correspond to providing meaningful benefits for the maturing years. The key to the success of such an approach is the capacity to start treatment perhaps as young as 4–5 years old, which is typically not the current practice with steroids. As mentioned earlier, DNP induces BDNF in the brain, but BDNF is also a myokine outside of the brain [150]. This may provide some benefits to the muscles, including cardiac, and may also improve cognitive issues associated with DMD simultaneously [151,152].

## 20. Diabetes, Metabolic Syndrome and Fatty Liver Diseases

Given the number of drugs in the toolbox for diabetes relative to what is available for truly insidious neurodegenerative diseases, the latter is likely a better application initially for the introduction of mitochondrial uncouplers in the clinic. However, it is pretty clear that there is an application for metabolic diseases. It is a common finding that when energy expenditure increases even at very low levels, whole body flux of glucose improves by clearing of intra-hepatic and intra-muscular lipids [153,154,155,156,157,158,159]. This finding has applications to the plethora of co-morbidities and co-mortalities associated with the over-nutritional phenotype [2]. It was shown in humans, that even modest weight loss of ~8 lbs can have a profound effect on improving metabolic endpoints [160]. Diet, including caloric restriction and exercise (ideally combined), is a prudent first line of treatment [161]. Focusing on the energy-out side of the equation, one other possible approach is to utilize DNP’s pharmacology, or modified forms of DNP, in low doses to increase energy expenditure [2,84]. Although our understanding of diabetes, insulin resistance and fatty liver diseases in the 1930s has greatly improved over 80 years, it was however reported even back then that for patients that did have diabetes and took DNP for long periods of time, that there was a significant improvement in lowering the AUC during an oral glucose tolerance test (OGTT) [162]. It was also reported that there was no improvement in an OGTT for obese individuals taking DNP for short durations. This makes sense now because it takes time to clear away intra-hepatic and intra-skeletal muscle lipids, from these two, key insulin-sensitive tissues needed to restore insulin sensitivity. This approach is completely different than the immediate effects of an insulin injection to drop blood glucose levels, but it has a far more profound long-term effect of potentially eliminating insulin injections altogether or eliminate the portfolio of anti-diabetic drugs taken for non-insulin dependent diabetic patients [2]. It seems surprising today, why in the 1930s the regime was to dose up to ~3 lbs/week, as in theory a much lower dose of ~50 mg/day would result in ~0.5 lbs of weight loss per week, corresponding to a dramatic ~26 lbs per year alone. However, weight loss may not be necessary to significantly improve many of the detrimental effects of the over-nutritional phenotype [2]. For example, in a study conducted in a colony of wildtype mice, provided a very low dose of DNP of ~100 μg/kg/day (HED ~0.5 mg/day) for basically their entire lifespan, resulted in lower circulating glucose, insulin and triglycerides and increased lifespan relative to placebo (water) [40]. The mice also had less ROS production, lower oxidized proteins and DNA damage in the brain, liver and cardiac tissues. Again, this speaks to the possible anti-aging properties of low dose uncouplers over long periods of time.

Samuel, V.T., Liu, Z.X., Qu, X., Elder, B.D., Bilz, S., Befroy, D., Romanelli, A.J., and G. I. Shulman (2004), provided rats a high fat diet for 3-days for preferential induction of hepatic insulin resistance without significant accumulation of lipids in the skeletal muscle. Simultaneously the rats were also provided 3-days of 16 mg/kg of DNP daily systemically, which had a striking effect on preventing hepatic fat accumulation vs. placebos [45]. The hyperinsulinemic-euglycemic clamp study showed that DNP treatment had a significant preservation of insulin-mediated suppression of endogenous glucose production. This is important since overt gluconeogenesis is a hallmark in most type 2 diabetics in the face of relatively flat glycogenolysis [163]. Perry, R.J., Kim, T., Zhang, X.M., Lee, H.Y., Pesta, D., Popov, V.B., Zhang, D., Rahimi, Y., Jurczak, M.J., Cline, G.W. et. al. (2013), made a modified version of DNP with a methyl-ether linker off the hydroxyl group (DNPME) that would allow for the molecule to get flagged by the p450’s in the liver and potentially sequestered to the liver [164]. The general idea was to improve the safety index by providing local delivery of DNPME to the liver. Then p450s would metabolize DNPME back to the active form of DNP, raise energy expenditure in the local hepatic environment, clear the lipids and restore hepatic insulin sensitivity. The rats were provided with a high-fat diet with sucrose in the drinking water to induce steatosis and then provided daily doses of DNPME for 5-days. The results showed lower fasting plasma glucose, triglyceride, and insulin concentrations compared to the vehicle-treated animals, without a change in body weight or food consumption. With specific delivery from the gut with the first pass through the liver from the portal vein, the safety index was improved. The treated rats also showed an improvement in skeletal muscle insulin resistance, so there may have been some DNP leaking out of the liver and/or cleaning up the liver had a cascade effect of reducing skeletal muscle lipids as well [164]. Liver-specific delivery of DNP does put a burden on an organ ~20% of the total human mass but may serve the purpose as a treatment for NASH [165]. Most over-nutritional individuals, both the liver and skeletal muscles show insulin resistance, as the adipose tissue threshold is exceeded for normal physiological function to sequester lipids [166]. In a follow-up, to the liver-specific delivery idea, Perry, R.J., Zhang, D., Zhang, X.M., Boyer, J.L., and G. I. Shulman (2015) developed a nanoparticle encapsulation of DNP as a slow release formulation [167]. So instead of using 20% of the body mass to clear lipids only from the liver, DNP would be provided systemically, similar to DNP provided to the colony of mice via the drinking water, which utilized ~100% of the body mass to improve whole body flux [40,167].

## 21. Concluding Thoughts

**Figure 7:**
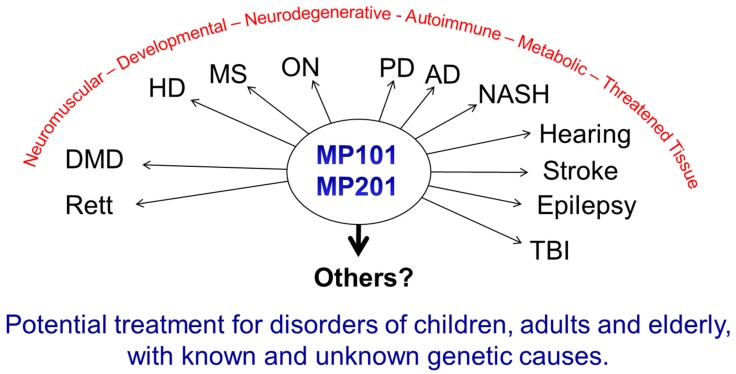
DNP as a Broad-Spectrum Treatment. DNP (MP101) or the prodrug of DNP (MP201) has been tested in disease models of acute and chronic studies with a known and unknown genetic cause that representing pediatric, adult and elderly indications with statistically positive outcomes. The indications also present diseases of neuromuscular disorders, development, neurodegeneration, autoimmune, metabolic and trauma. The future will help to determine the limitations of the pharmacology, but given the findings, it appears that DNP may be a broad-spectrum treatment to many disorders.

Moderate exercise induces BDNF elevation in the brain, as well as improves muscle tone, lowers lipids in key tissues for insulin sensitivity, reduces resting heart, blood pressure and general fitness [69,168,169]. Similar to the benefits of exercise, caloric restriction or fasting can have a positive effect on increased mitochondrial bioenergetics and mitochondrial stress by up-regulation of CREB, BDNF, autophagy, PGC-1A, and sirtuin 3 while lowering mTOR signaling (apoptosis) in the hippocampus and other regions of the brain [161,170,171]. The impact of fasting applied to patients appears to improve a host of endpoints related to neurodegeneration, aging and epilepsy, even tolerance to the side effects of chemotherapy [40,52,170,172,173,174,175,176]. Therefore, if this is possible, the arrows point towards the mitochondria as a central point and rationale target as an intervention point on a plethora of diseases. Although diet and exercise should always be the first line of defense, DNP appears to mimic in part the neuroprotective and neurorestorative effects of exercise and fasting by increasing BDNF, lowering cellular stress and building cellular resiliency by mild increases in mitochondrial bioenergetics. BDNF production via CREB is known to be induced by increases of second messenger, cAMP and Ca^2+^ [177,178]. Primary rat hippocampal cell cultures treated with 20 μM DNP increases production of cAMP [27]. The mechanism of action for the increase is unknown, but it is likely due to the activation of adenylate cyclase (AC) that synthesizes ATP into cAMP in the cytosol when DNP lowers the mitochondrial membrane potential. This causes a lower influx of Ca^2+^ into the matrix via closing the uniporter, but simultaneously a rise in the cytosolic Ca^2+^ (see Figure 1) [30]. There are nine mammalian transmembrane AC (tmACs) and one soluble isoform (sAC), which is associated with the mitochondria, neurite growth and directly activated by calcium independently of calmodulin [179]. Isoform I, III and VIII AC are stimulated by Ca^2+/^calmodulin, whereas V and VI are inhibited by Ca^2+^ [180]. It is possible that the increase in cAMP is also due to an inactivation of phosphodiesterases (PDEs) that degrades cyclic nucleotides as well. PDE inhibitors have been sought after to improve cognition, reduce episodes of schizophrenia, depression and improve mental stability with the rise of cAMP, which DNP treatment may address as well [181,182]. The collective benefits of DNP driven increases of cAMP, CREB and BDNF may be vast for mental health. Treatment of animals representing a myriad of human neurodegenerative diseases with DNP appears pro-neuroprotective for diseases of known and unknown etiology, and indications of all ages (pediatrics, adult and elderly). It is not a “magical elixir”, but based upon the scientific fact that: 1) all human cells have mitochondria except mature red blood cells, 2) they have a symbiotic relationship within the cell governing cell survival and functions that impact many pathways, and 3) from experimental studies in so many laboratories across the world that have compiled both positive and significant data showing its merit in animal models of Huntington Disease, Parkinson Disease, Alzheimer’s Disease, Multiple Sclerosis, Rett Syndrome (data not shown), epilepsy, hearing loss, vision loss (optic neuritis), traumatic brain injury, and Duchenne Muscular Dystrophy [28,52,85,86]. Others have tested DNP in models of stroke, sciatic nerve injury, TBI, Aβ_1-42_ inhibition of plaque formation and various metabolic diseases [29,45,87,88,164,167,183]. This spectrum corresponds to diseases of developmental (Rett), neuromuscular (DMD), metabolic (NASH, diabetes, insulin resistance), neurodegeneration (AD, PD, HD, etc.), autoimmune (MS, ON, etc.) and trauma (hearing, stroke, nerve damage, TBI) (Figure 7).

Collectively, DNP may be a treatment for an emerging global term called “metabesity” referring to all the co-morbidities associated with the over-nutritional phenotype such an increased incidence of insulin resistance, obesity, type 2 diabetes, sleep apnea, depression, inflammation, cardiovascular disease, hypertension, non-alcoholic fatty liver disease, but includes accelerated aging, neurodegeneration, and cancer. [2,184,185]. Studies are underway to explore an ever-expanding application of low dose mitochondrial uncoupling pharmacology. There are other insidious diseases like Pompe, Wolfram Syndrome, Friedreich ataxia, cardiolipin production, etc., that are associated with mitochondrial dysfunction of Ca^2+^, ATP and ROS homeostasis, that have no cures, so it is important to understand if DNP or modified versions of DNP may have merit in these diseases as well [15,43,46,186,187,188]. Since DNP was tested in many laboratories, for completely diverse indications, it speaks to the idea that modulating mitochondrial physiology with uncouplers, can enlist cellular resiliency that is global to many diseases. Therefore, when it comes to mulling on whether a particular disease has a mitochondrial component and whether or not DNP could have merit, it is imperative that it just gets tested.

**Figure 8:**
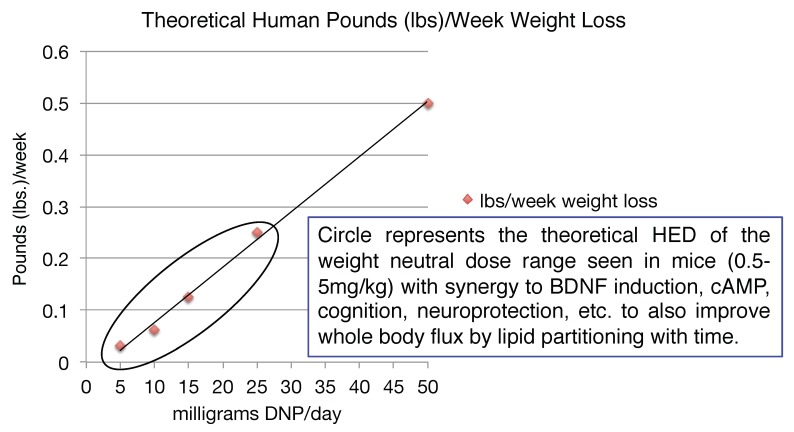
Paradigm shift from weight loss program to a wellness program. Extrapolating the human published data from the 1930s to doses that are significantly lower and likely within the hormetic range of inducing BDNF to gain synergy, while simultaneously partitioning lipids out of insulin-sensitive tissues at weight neutral doses. Human equivalent dose (HED) within this range offers the possibility to significantly improve whole body flux at safe doses using time as the primary variable.

Related to metabolic disease, perhaps it is time for a paradigm shift from the lofty goals of weight loss, to a wellness program. Merging the 1930s data that suggested long chronic treatment can lower the AUC during an oral glucose tolerance test (OGTT) in obese individuals (but not short durations), with the idea that very low doses over long periods of time can have a striking effect on many endpoints, could be such a paradigm shift. The metabolic impact of raising energy expenditure by a small degree to partition fat out of the insulin-sensitive tissues (liver and muscle), could, in fact, emerge as a method of treatment for the intractable over-nutritional phenotype at safe, weight *neutral* doses (Figure 8) [2,189]. Since weight neutral doses of DNP significantly raises BDNF, which also has anti-diabetic properties in peripheral organs, there may be a synergistic effect of clearing lipids while raising BDNF [70,71,72,73,190]. This approach is significantly safer than the approach in the 1930s, as doses would be at least 10–60× lower [52]. Another possible approach is to use a prodrug of DNP with extended residency time. The possible advantage with this strategy vs. DNPME or any organ-specific delivery is that DNP circulates systemically to utilize the whole-body mass, requiring even lower doses. Whole body insulin sensitivity should improve while increasing the safety index with slow absorption and lower C_max_. Time will be the major variable as to when insulin sensitivity would be restored, however, it took a long time to create the issue with over-nutrition, therefore, it will require time to resolve safely. The impact could be profound on the metabolic syndrome with doses within the low hormetic range and perhaps simultaneously even increase cognition, or prevent cognitive decline in aging populations. A likely well-overdue breakthrough approach.

DNP is not known for inherent addictive properties like a narcotic or amphetamine, except for the allure of slimming down and quickly. Therefore, the concerns over whether or not DNP today would get abused for neuroprotection as in the 1930s for obesity is damped by the data that low doses have a major impact in the animal models listed here and there is a hormetic effect in that “less is more” for optimal impact on disease progression. The dose window that we have seen with all the animal studies has been between ~0.5–5 mg/kg in mice, which correlates to a HED range between ~2–22 mg/day. If this translates to the human scenario, including the hormetic response, then it would not make sense to push the dose for instance to 22 mg for AD, which corresponds to 5 mg/kg in the mouse model, when it was clearly shown that 0.5 mg/kg or less in the APP/PS1 was optimal, a HED of 2 mg/day or lower. Rarely does a mouse efficacious dose curve translate perfectly well to humans, but since mitochondria are extremely well evolutionarily conserved from mouse to humans, and the effect is non-genomic (not relying on the conservation of a protein or receptor from mouse to human), then the prediction of translation is excellent relative to other platforms. Finding the optimal dose however will likely become the costliest aspect of clinical development, as it is the normal practice that when a dose does not meet its critical endpoint, the dose is escalated higher. In this case, however, it may be beneficial to lower the dose. Since only under chronic treatment will the full pharmacology be onboard with the rise of BDNF, we (Mitochon Pharma.) have made it a general mandate to test in vivo with once-per-day oral administration. If we rank ordered indications from insidious to less insidious, diabetes would be lower on the list, even though there is a likely application. We can learn however from the possible clinical studies of diseases like Wolfram Syndrome (DIDMOAD), Rett Syndrome, Friedreich ataxia, or Huntington’s disease that are associated with diabetes or insulin resistance, if there is an improvement in this secondary or tertiary symptoms [15,191,192,193]. The primary endpoint, of course, would be to block neurodegeneration, but improvements in metabolic flux could also be monitored. In particular, there may be an application on multiple fronts with diseases like Wolfram Syndrome. Wolfram Syndrome is an ER Ca^2+^ stress condition that starts with juvenile diabetes, and progresses into vision loss, hearing loss and both voluntary and involuntary muscle loss without the loss of cognition until death ~20–30 years of age. The mutation is not caught at the diabetes stage but typically diagnosed during vision loss. Here perhaps, DNP may have an application since prior studies have protected against vision loss in the optic neuritis model, hearing loss in the noise trauma model, lowering of calcium overload in models of TBI and neuroprotection in a host of models. Insulin sensitivity could also be improved but of less of a concern. Similar in chronic treatment of Huntington’s patients, metabolic improvements would be a tertiary endpoint but could be monitored and likely improved with low doses intended over a long period of time [194]. The primary endpoint beyond safety and tolerability would be improving motor function (Total Motor Score), cognitive function (UHDRS), global clinical status (CGI) and preservation of brain volume by MRI.

The progress that has been made in the science of mitochondrial bioenergetics has been tremendously important to gain an understanding of the central role in the cell that this organelle governs over so many pathways. The acceleration of mitochondrial research in the last couple of decades and an emerging focus on the legion of diseases is remarkable. With over 80 years since DNP has been in a clinical study, there is also a tremendous opportunity to learn today what merits the pharmacology may provide repositioned for truly insidious diseases using modern day clinical practices. This approach is a much lower hanging fruit than the very high hurdles of gene replacement as a first line of attack. It is possible to wake up redundant cellular compensatory mechanisms by modulating the mitochondria’s entire physiology towards pro-survival of the cell, with a brain penetrant, simple oral dosing of DNP to attenuate disease progression for a broad number of indications, slow aging, and the potential in the future to prophylactically treat patients to entirely prevent the myriad of age-related illnesses. It may sound unorthodox, but it falls within the physiology/pharmacology of the mechanism of action to be plausibly *true*.

## Supplementary Materials

The following are available online https://www.mdpi.com/2073-4409/8/3/280/s1. Movie S1 MP101 and MP201 Prevents Paralysis in EAE Model.
